# Clonotype specificity distinguishes epitope-tailored and permissive somatic hypermutation

**DOI:** 10.64898/2026.10.10.758199

**Published:** 2026-10-16

**Authors:** Vishal N. Rao, Bruno Bonnettaz, Alesandro Civljak, Anass Abbad, Florian Krammer, Camila H. Coelho, Goran Bajic

## Abstract

Identifying antibody features that reliably encode epitope specificity poses a major barrier towards modeling antigen-antibody interactions. Affinity maturation offers a unique opportunity to probe the molecular basis of antigen-antibody interactions by revealing how individual somatic hypermutations (SHM) refine antibody binding. Using SARS-CoV-2 spike-specific germinal center B-cell repertoires, we show that antibodies harboring multiple SHMs that are restricted to their respective clonotypes (clonotype-restricted SHMs) preferentially recognize cryptic epitopes. We structurally characterized three such antibodies: two recognized a cryptic epitope on the receptor-binding domain whereas a third bound an N-terminal domain epitope that is almost entirely occluded in prefusion spike. In contrast, SHMs shared across multiple clonotypes using the same V gene recur across antibodies targeting diverse antigens but generally fail to enhance affinity when introduced into diverse antibody backgrounds. Together, these findings define a spectrum of SHM behavior, distinguishing epitope-tailored mutations from broadly recurrent but largely permissive substitutions.

**One Sentence Summary:**

The extent to which a somatic hypermutation is shared across antibody clonotypes distinguishes epitope-tailored mutations associated with cryptic epitope recognition, from recurrent, largely permissive substitutions.

## Full Text Availability

The license terms selected by the author(s) for this preprint version do not permit archiving in PMC. The full text is available from the preprint server.

